# Longitudinal Predictors of Informant-Rated Involvement of People with Dementia in Everyday Decision-Making: Findings from the IDEAL Program

**DOI:** 10.1177/07334648221128558

**Published:** 2022-10-04

**Authors:** Serena Sabatini, Anthony Martyr, Laura D. Gamble, Rachel Collins, Fiona E. Matthews, Robin G. Morris, Jennifer M. Rusted, Claire Pentecost, Catherine Quinn, Linda Clare, behalf of the IDEAL study team

**Affiliations:** 1 Faculty of Biomedical Sciences, 27216 University of Italian Switzerland, Lugano, Switzerland; 2 Centre for Research in Ageing and Cognitive Health, 3286 Faculty of Health and Life Sciences, University of Exeter Medical School, Exeter, UK; 3 Population Health Sciences Institute, Newcastle University, Newcastle, UK; 4 Department of Psychology, King’s College London, Institute of Psychiatry, Psychology and Neuroscience, London, UK; 5 School of Psychology, 1948 University of Sussex, Brighton, Brighton, UK; 6 Centre for Applied Dementia Studies, Bradford University, Bradford, UK; 7 Wolfson Centre for Applied Health Research, Bradford, UK

**Keywords:** Alzheimer’s disease, caregiving, functional ability, cognitive capacity, neuropsychiatric symptoms, caregiver stress, relationship quality

## Abstract

The extent to which people with dementia are involved in everyday decision-making is unclear. We explored informant-rated involvement of people with dementia in everyday decision-making over 2 years and whether functional, behavioral, and psychological factors related to the person with dementia and the caregiver explain variability in involvement of people with dementia in everyday decision-making. We used IDEAL data for 1182 people with dementia and their caregivers. Baseline mean score on the decision-making involvement scale was 31/45; it minimally declined over time. People with dementia who were female, single, and/or whose caregiver was younger had greater involvement in everyday decision-making than those without these characteristics. Better cognition, fewer functional difficulties, fewer neuropsychiatric symptoms, less caregiver stress, and better informant-rated relationship quality were associated with higher involvement in everyday decision-making. Cognitive and functional rehabilitation, and educational resources for caregivers, could prolong involvement of people with dementia in everyday decision-making.

**What this paper adds**

• According to their caregivers, people with mild-to-moderate dementia are involved in everyday decision-making but their involvement declines over time.
• People with dementia who are female, single, and/or whose caregiver is younger are more involved in everyday decision-making compared to those who do not share these characteristics.
• Better cognitive and functional abilities and fewer neuropsychiatric symptoms in people with dementia, less caregiver stress, and better informant-rated relationship quality are associated with higher involvement of people with dementia in everyday decision-making.

**Applications of study findings**

• As the severity of dementia progresses, people with dementia are less involved in everyday decision-making. Carers may benefit from educational resources that teach them strategies to continue involving people with dementia in everyday decision-making as the illness progresses.
• As people with dementia who are better educated are more involved in everyday decision-making, strengthening education, for example, through lifelong continuous education programs, may support adjustment to the onset of dementia, including maintenance of involvement in decision-making.
• Those carers who take more everyday decisions on behalf of the person with dementia report higher stress and may therefore benefit greatly from psychological support.

## Introduction

Globally, 55 million people are living with dementia (World Health Organization, 2021). Dementia involves a progressive decline in cognitive and functional abilities (American Psychiatric Association, 2013; Martyr & Clare, 2012; Suh et al., 2004; World Health Organization, 2018), leading to assumptions that people with dementia are unable to make certain decisions (Miller et al., 2014). Decisions range in complexity and importance, from decisions about current and/or future care to everyday decisions such as what to eat, what to wear, and when to go to bed (Delazer et al., 2007; Menne et al., 2008; Sinz et al., 2008). In fact, people with early-stage dementia are generally able to make most decisions (Davis et al., 2017; Feinberg & Whitlatch, 2001) and people in the later stages of dementia, who are still able to communicate preferences, may be able to make simpler everyday decisions. Many people with mild-to-moderate dementia want greater involvement in decision-making (Fetherstonhaugh et al., 2013; Hirschman et al., 2005; Whitlatch & Menne, 2009). However, one qualitative study involving interviews with 12 people with dementia and their caregivers on four occasions over 1 year suggests that as dementia severity increases people with dementia become gradually less involved in everyday decision-making (Samsi & Manthorpe, 2013).

The United Nations Convention on the Rights of Persons with Disabilities (Nations, 2022) sets out the rights of people with disabilities, including dementia, to make their own decisions wherever possible. Many countries have introduced legislation to support and safeguard these rights; examples include the Mental Capacity Act in England and Wales (Ministry of Justice, 2007), the Adults with Incapacity Act in Scotland (The Department of Health, 2000), and the Health Care Consent Act in Canada (Health Care Consent Act, 1996). Being involved in decision-making is important for people with dementia as it enables and preserves a sense of autonomy and control over life and contributes to a good quality of life (Bonds et al., 2021; Fetherstonhaugh et al., 2013; Menne et al., 2002, 2008, 2009; Menne & Whitlatch, 2007). This need for greater involvement remains central to decisions that affect them, such as planning ahead for when they are unable to make decisions about care (Fetherstonhaugh et al., 2013), and decisions around current treatment and care (Hirschman et al., 2005). Most research on decision-making has focused on how involved people with dementia are in major life decisions such as driving cessation (Adler, 2010), medical treatment, moving to nursing or care facilities (Thein et al., 2011), and end-of-life care (Caron et al., 2005). Less is known about the degree of involvement of people with dementia in everyday decision-making and how this changes over time.

Involvement in everyday decision-making of people with dementia may be related to a range of personal characteristics of the person with dementia and caregiver. Indeed, a study reported that people with dementia were more involved in everyday decision-making if they were younger, female, had more education, had fewer functional limitations, and had a non-spousal caregiver (Menne & Whitlatch, 2007). This study was cross-sectional and there is therefore now a need for longitudinal evidence exploring whether involvement of people with dementia in everyday decision-making decreases as cognitive, physical, and functional difficulties increase. It may also be that people with certain diagnostic subtypes, such as those with frontotemporal dementia, are perceived as having more impaired reasoning and this may deter their caregivers from involving them in everyday decision-making (Piguet et al., 2011). The link between dementia subtype and involvement in everyday decision-making has, to our knowledge, never been explored.

Characteristics of the caregiver, such as caregiver stress and perception of the relationship quality with the person with dementia, may also be related to the extent to which caregivers involve the person with dementia in everyday decision-making. Indeed, those caregivers who experience their role more positively, report less stress, and/or have a higher-quality relationship with the person with dementia provide better care for the person with dementia (Chunga et al., 2021; Quinn et al., 2020) and this may include greater efforts to ensure involvement in everyday decision-making.

This study aims to (1) describe levels of informant-rated (i.e., rated by the caregiver) involvement of people with dementia in everyday decision-making at baseline and 12 and 24 months later; (2) explore whether person with dementia (i.e., age, sex, dementia subtype, education, marital status, and living situation) and caregiver (i.e., age, sex, and caregiver status) factors are related to how involved people with dementia are in everyday decision-making at baseline; and (3) estimate change in everyday decision-making over time and its longitudinal associations with cognitive capacity, functional ability, neuropsychiatric symptoms, relationship quality, and caregiver stress.

## Methods

This study used data collected at baseline (T1: 2014–16); 12-month follow-up (T2: 2015–17); and 24-month follow-up (T3: 2016–18) of the IDEAL study. Study analyses used version six of the datasets. IDEAL participants were recruited through a network of 29 National Health Service (NHS) sites in England, Scotland, and Wales. Participants of any age could take part in IDEAL if they lived in the community, had a diagnosis of any type of dementia, and had mild-to-moderate cognitive decline (Mini-Mental State Examination score ≥ 15) (Folstein et al., 1975) at enrollment. Potential participants were excluded if, at baseline, they had a co-morbid terminal illness, were unable to provide informed consent, and/or there was known potential for home visits to pose risk to research staff. When a person with dementia joined the study, an informal caregiver was invited to participate, where available. There were no specific inclusion or exclusion criteria for caregivers. Further information about the IDEAL programme is reported in the study protocol (Clare et al., 2014). The IDEAL study was approved by the Wales five Research Ethics Committee (reference: 13/WA/0405) and the Ethics Committee of the School of Psychology, Bangor University (reference: 2014–11684). The IDEAL study is registered with the UK Clinical Research Network (registration number: 16593).

### Measures

#### Informant-rated measures

***everyday decision-making*** was assessed at all three timepoints with the 15-item Decision-Making Involvement Scale (Menne et al., 2008); see Supplementary Table 1 for details. Higher scores (range: 0–45) indicate greater involvement.

***Functional ability*** was assessed at all three timepoints with an amended informant-rated 11-item version of the Functional Activities Questionnaire including an additional question concerning appropriate telephone use (Martyr & Clare, 2012; Martyr et al., 2019; Pfeffer et al., 1982). Higher scores (range: 0–33) indicate greater functional limitations.

***Neuropsychiatric symptoms*** were assessed at all three timepoints with the Neuropsychiatric Inventory Questionnaire (Kaufer et al., 2000; Morris & National Alzheimer's Coordinating Center, 2008). The questionnaire covers 12 neuropsychiatric symptoms comprising sleep, apathy, delusion, depression, anxiety, euphoria, agitation, eating/appetite, hallucination, disinhibition, irritability, and aberrant motor behavior. The total score (range: 0–12) indicates the number of neuropsychiatric symptoms the person with dementia has.

#### Measures self-rated by the caregiver

##### Stress

was assessed with the 15-item Relative Stress Scale (Greene et al., 1982) at all three timepoints. Higher scores (range: 0–60) indicate greater stress.

##### Personal characteristics of the caregiver

comprised age, age groups (<65; 65–69; 70–74; 75–79; 80+), sex, and caregiver status (spouse/partner; family/friend).

#### Measures self-rated by the person with dementia

##### cognitive capacity

was assessed at all timepoints with the Addenbrooke’s Cognitive Examination-III (Hsieh et al., 2013). Higher scores (range: 0–100) indicate better cognitive capacity.

##### Personal characteristics of the person with dementia

comprised age, age groups (<65; 65–69; 70–74; 75–79; 80+), sex, education, marital status, living situation, and diagnosis subtype. Education comprised four groups: no qualifications; school leaving certificate at age 16; school leaving certificate at age 18; and university. Marital status comprised four categories: single; married/remarried/in a civil partnership; divorced/separated; widowed and not remarried. Living situation comprised four groups: living alone; living with spouse/partner; living with others; and living in care. This last group was applicable only at 12-months and 24-months as some people with dementia moved into residential care during the course of the study, and their caregivers remained in the study. Diagnosis subtype comprised seven groups: Alzheimer’s disease; vascular dementia; mixed-Alzheimer’s disease and vascular dementia; frontotemporal dementia; Parkinson’s disease dementia; dementia with Lewy bodies; and unspecified/other.

#### Measures rated by both the informant and the person with dementia

##### Quality of the relationship

between the person with dementia and the caregiver was assessed at all three timepoints with a modified version of the Positive Affect Index comprising five questions addressing communication quality, closeness, similarity of views on life, engagement in joint activities, and overall relationship quality (Clare et al., 2012). Higher scores (range: 5–30) indicate better relationship quality.

### Statistical Methodology

Descriptive statistics for study variables at all timepoints were reported.

Univariable regression models were conducted to explore, at baseline, whether characteristics of the person with dementia (age, sex, dementia subtype, education, marital status, and living situation) and of the caregiver (age, sex, and caregiver status) explained a significant amount of variance in scores on the decision-making involvement scale. A multiple regression model comprising all these predictors was also conducted.

Unadjusted and adjusted (age, sex, and diagnosis subtype of the person with dementia) latent growth curve models explored within- and between-individual differences in baseline levels of informant-rated everyday decision-making, and the trajectory of change in informant-rated everyday decision-making over the three timepoints.

Latent growth curve models investigated whether cognitive capacity, informant-rated functional ability, informant-rated neuropsychiatric symptoms, self-rated relationship quality between the person with dementia and the caregiver, caregiver stress, and informant-rated relationship quality were time-varying predictors of scores on the decision-making involvement scale over time. Each model estimated the concurrent associations between the selected predictor at baseline and everyday decision-making at baseline, the selected predictor at 12-months follow-up and everyday decision-making at 12-months follow-up, and the selected predictor at 24-months follow-up and everyday decision-making at 24-months follow-up. Unadjusted and adjusted (age, sex, and diagnosis subtype of the person with dementia) models were fitted. All latent growth curve models had good model fit indices (Comparative Fit Index/Tucker-Lewis Index > .95, Root Mean Square Error of Approximation < .05).

Univariate regression models were conducted using Stata whereas latent growth curve models were conducted using Mplus (Muthén & Muthén, 1998-2017; StataCorp, 2017). For predictors, missing data were imputed using multiple imputation by chained equations, generating 25 imputed datasets. Missing data on the outcome measure was handled using maximum likelihood estimation with robust standard errors.

## Results

### Descriptive Statistics

Of the 1545 people with dementia that took part in IDEAL, 268 were excluded from study analyses as they did not have a caregiver taking part. A further 29 participants were excluded as their caregiver changed over the study period which precluded comparison of change in scores over time. An additional 66 participants were excluded as they had missing data for everyday decision-making at all timepoints. The analyses therefore comprised 1182 people with dementia at baseline, 948 at 12-month follow-up, and 707 at 24-months follow-up.

Descriptive statistics for study variables at all timepoints are reported in Table 1. Mean age of people with dementia was 76.10 years (SD = 8.17) at baseline. Slightly above half were men (59.1%), most were married or co-habiting with a partner, and just over half had a diagnosis of Alzheimer’s disease. According to their caregivers, people with dementia generally had moderate levels of functional impairment and three to four neuropsychiatric symptoms. People with dementia rated the quality of their relationship with the caregiver positively. Characteristics of people with dementia at follow-ups were similar to baseline but functional impairment increased slightly over time.

**Table 1.** Personal Characteristics at baseline, 12-month and 24-month follow-ups.

	Baseline (n = 1182)	12-month follow-up (*n* = 948)	24-month follow-up (*n* = 707)
Measures self-rated by the person with dementia			
Age in years, M (SD; range)	76.10 (8.17)	76.80 (8.00)	77.19 (8.02)
Age group, *n* (%)			
Aged < 65	95 (8.0)	65 (6.9)	50 (7.1)
Aged 65–69	147 (12.4)	111 (11.7)	67 (9.5)
Aged 70–74	217 (18.4)	178 (18.8)	144 (20.4)
Aged 75–79	286 (24.2)	217 (22.9)	147 (20.8)
Aged ≥ 80	437 (37.0)	377 (39.9)	299 (42.3)
Sex, *n* (%)			
Women	484 (41.0)	385 (40.6)	290 (41.0)
Men	698 (59.1)	563 (59.4)	417 (59.0)
Education, *n* (%)			
No qualification	315 (26.7)	244 (25.7)	183 (25.9)
Leaving certificate at age 16	211 (17.9)	168 (17.7)	119 (16.8)
Leaving certificate at age 18	408 (34.5)	337 (35.6)	252 (35.6)
University	243 (20.6)	197 (20.8)	152 (21.5)
Missing	5 (0.4)	2 (0.2)	1 (0.1)
Marital status, *n* (%)			
Single	12 (1.0)	7 (0.7)	6 (0.8)
Married/partner/co-habiting	977 (82.7)	781 (82.4)	576 (81.5)
Divorced/separated	47 (4.0)	32 (3.4)	21 (3.0)
Widowed	146 (12.4)	106 (11.2)	66 (9.3)
Missing, *n* (%)	0 (0)	22 (2.3)	38 (5.4)
Living situation, *n* (%)			
Living alone	132 (11.2)	84 (9.1)	46 (7.1)
Living with spouse/partner	987 (83.4)	779 (84.1)	556 (85.4)
Living with other	61 (5.2)	42 (4.5)	29 (4.5)
Living in care	0 (0)	20 (2.2)	20 (3.1)
Missing, *n* (%)	2 (0.2)	23 (2.4)	56 (7.9)
Diagnosis subtype, *n* (%)			
Alzheimer’s disease	659 (55.8)	529 (55.8)	407 (57.6)
Vascular dementia	122 (10.3)	89 (9.4)	67 (9.5)
Mixed (Alzheimer’s and vascular)	249 (21.1)	208 (21.9)	149 (21.1)
Frontotemporal dementia	45 (3.8)	37 (3.9)	30 (4.2)
Parkinson’s disease dementia	39 (3.3)	32 (3.4)	17 (2.4)
Dementia with lewy bodies	38 (3.2)	32 (3.4)	21 (3.0)
Unspecified/other dementia	30 (2.5)	21 (2.2)	16 (2.3)
Cognitive capacity, M (SD)	69.03 (13.40)	65.54 (16.19)	63.55 (18.43)
Missing, *n* (%)	69 (5.8)	96 (10.1)	111 (15.7)
Relationship quality; M (SD)	25.12 (3.55)	25.06 (3.65)	25.15 (3.66)
Missing, *n* (%)	148 (12.5)	83 (8.8)	120 (17.0)
Measures informant-rated by the caregiver			
Functional ability, M (SD)	17.82 (8.60)	20.92 (8.63)	22.91 (8.76)
Missing, *n* (%)	70 (5.9)	39 (4.1)	21 (3.0)
Number of neuropsychiatric symptoms, M (SD)	3.57 (2.45)	3.74 (2.52)	4.16 (2.53)
Missing, *n* (%)	47 (4.0)	35 (3.7)	30 (4.2)
Everyday decision-making, M (SD)	31.10 (10.0)	29.22 (11.21)	26.96 (12.32)
Missing, *n* (%)	64 (5.4)	54 (4.3)	35 (5.0)
Measures self-rated by the caregiver			
Age in years, M (SD; range)	69.05 (10.90)	70.30 (10.51)	71.07 (10.33)
Age group, *n* (%)			
Aged < 65	338 (28.6)	242 (25.5)	152 (21.5)
Aged 65–69	200 (16.9)	145 (15.3)	116 (16.4)
Aged 70–74	244 (20.6)	207 (21.8)	162 (22.9)
Aged 75–79	212 (17.9)	176 (18.6)	129 (18.3)
Aged ≥ 80	188 (15.9)	178 (18.8)	148 (20.9)
Sex, *n* (%)			
Women	822 (69.5)	657 (69.3)	485 (68.6)
Men	360 (30.5)	291 (30.7)	222 (31.4)
Caregiver status, *n* (%)			
Spouse/partner	969 (82.0)	799 (84.3)	601 (85.0)
Family/friend	213 (18.0)	149 (15.7)	106 (15.0)
Stress, M (SD; range)	19.09 (9.85)	21.74 (10.08)	23.05 (10.19)
Missing, *n* (%)	52 (4.4)	65 (6.9)	45 (6.4)
Relationship quality, M (SD; range)	23.16 (4.71)	22.23 (4.87)	21.84 (4.80)
Missing, *n* (%)	18 (1.5)	53 (5.6)	37 (5.2)

M = mean. SD = standard deviation. *n* = number.

At baseline, caregivers’ mean age was 69.05 years (SD = 10.90). Over two-thirds of caregivers were women. The majority (82%) were spouses or co-habiting partners of the person with dementia. Caregivers self-reported relatively low levels of stress and rated the quality of their relationship with the person with dementia positively.

Mean decision-making involvement scale score was 31.10; hence caregivers generally reported that people with dementia were “fairly involved” or “very involved” in the everyday decisions that are included in the decision-making involvement scale. Number and proportions of caregivers’ responses for the decision-making involvement scale items at all timepoints are reported in Supplementary Table 1. At baseline, caregivers reported that the person with dementia was most involved in making daily and personal decisions about when to go to bed (88.3% were very or fairly involved); when to get up (84.9% were very or fairly involved); medical care (79.9% were very or fairly involved), what to do in spare time (79.7% were very or fairly involved); and expressing affection (76.0% were very or fairly involved). However, fewer people with dementia were involved in decisions about places to go (66.8% were very or fairly involved); spending money (67.8% were very or fairly involved); and visiting friends (67.3% were very or fairly involved). Caregivers reported that a high proportion of people with dementia were involved in important decisions such as where to live (82.2% were very or fairly involved). According to caregivers, a lower proportion of people with dementia was involved in those decisions that may be relevant only for a subgroup of the older population such as doing physical activity (70.8% were very or fairly involved); having a pet (68.3% were very or fairly involved); and decisions around religion and spiritual activities (43.6% were very or fairly involved). Compared to other types of everyday decisions, involvement in decisions about what clothes to wear (68.6% were very or fairly involved); meals (66.0% were very or fairly involved); and what food to buy (57.2 were very or fairly involved) was lower.

In this sample, women were more involved than men in all types of decision-making (see Supplementary Table 2) but gender differences were more pronounced in decisions about what clothes to wear (72% of men vs. 91.6% of women were very or fairly involved), meals (56.2% of men vs. 79.9% of women were very or fairly involved), and what food to buy (46.5% of men vs. 72.6% of women were very or fairly involved).

### Associations between Characteristics of the Person with Dementia and of the Caregivers with Involvement of People with Dementia in Decision-Making

Table 2 reports regression models addressing associations between baseline person with dementia and caregiver characteristics and decision-making involvement scores. In the univariable model, younger age, being female, being unmarried, not living with a spouse/partner, and having a non-spousal caregiver were associated with greater involvement in everyday decision-making, as was having a male caregiver and a younger caregiver. When these characteristics were modeled together, associations remained for sex, marital status, and educational level of the person with dementia, dementia subtype, and age of the caregiver. People with dementia who were female and/or single had greater involvement in everyday decision-making. Those with frontotemporal dementia were less involved in everyday decision-making compared to those with Alzheimer’s disease. Those with a university degree were more involved in everyday decision-making than those with no education. Those caregivers who were younger in age reported greater involvement of the person with dementia in everyday decision-making.

**Table 2.** Regression models with everyday decision-making as outcome and characteristics of the person with dementia and of the caregiver as predictors at baseline.

	Univariable regressions		Multivariable regression	
Predictors at baseline	Beta (95% CI)	*p*-Value	Beta (95% CI)	*p*-value
Age of the person with dementia	−.11 (−.19; −.04)	.002	−.08 (−.20; .03)	.161
Sex of the person with dementia (Reference: male)	4.16 (2.99; 5.34)	<.001	3.16 (.86; 5.45)	.007
Dementia subtype (Reference: Alzheimer’s disease)				
Vascular dementia	1.03 (−1.00; 3.05)	.320	.93 (−1.01; 2.88)	.347
Mixed (Alzheimer’s and vascular)	−.12 (−1.62; 1.39)	.878	.08 (−1.38; 1.53)	.919
Frontotemporal dementia	−2.31 (−5.35; .73)	.136	−3.05 (−6.02; −.08)	.044
Parkinson’s disease dementia	.40 (−2.94; 3.73)	.815	.98 (−2.20; 4.17)	.545
Dementia with lewy bodies	−2.31 (−5.68; 1.07)	.180	−1.21 (−4.45; 2.03)	.464
Unspecified/other dementia	−.89 (−4.83; 3.05)	.658	−1.17 (−4.98; 2.63)	.545
Education (Reference: no education)				
School leaving certificate age 16	.06 (−1.74; 1.85)	.951	.516 (−1.23; 2.26)	.561
School leaving certificate age 18	−1.0 (−2.51; .51)	.194	.13 (−1.36; 1.63)	.862
University	.75 (−.98; 2.48)	.397	1.80 (.10; 3.50)	.038
Marital status (Reference: Married/Partner/Co-habiting)				
Single	7.80 (2.19; 13.01)	.006	7.40 (.00; 15.79)	.041
Divorced/separated	7.37 (4.45; 10.28)	<.001	4.08 (−.97; 9.08)	.113
Widowed	4.27 (2.50; 6.05)	<.001	2.07 (−.3.07; 7.21)	.430
Living situation (Reference: Spouse/Partner)				
Living alone	6.26 (4.42; 8.11)	<.001	1.59 (−4.47; 7.66)	.606
Live with other	2.83 (.20; 5.47)	.035	−2.09 (−8.30; 4.12)	.509
Age of the caregiver	−.19 (−.24; −.13)	<.001	−.15 (−.26; −.03)	.010
Sex of the caregiver (Reference: male)	−2.39 (−3.67; −1.12)	<.001	−.85 (−3.11; 1.41)	.461
Caregiver status (Reference: spouse)				
Family/Friend	4.84 (3.33; 6.35)	<.001	−1.32 (−6.18; 3.54)	.594

### Time-Varying Predictors of Involvement in Everyday Decision-Making

At baseline, for the overall cohort the mean decision-making involvement score was 31.22, and there was a decline of 2.71 points per year. Unadjusted and adjusted latent growth curve models explored change in everyday decision-making over time; see Table 3. In the adjusted model, better cognitive capacity, fewer functional difficulties, fewer neuropsychiatric symptoms, less caregiver stress, and better informant-rated relationship quality were associated with higher involvement in everyday decision-making. These associations increased in strength over time. In the adjusted model, self-rated quality of the relationship between the person with dementia and the caregiver was not associated with involvement in everyday decision-making at any timepoint.

**Table 3.** Latent growth curve models estimating change in everyday decision-making and time-varying predictors of decision-making.

	Decision-making involvement scale over time					
	Unadjusted Model			Adjusted Model		
Time-varying predictors	T1	T2	T3	T1	T2	T3
	(Estimate 95% CI)					
Functional ability at T1	−.21 (−.25; −.08)			−.51 (−.55; −.46)		
Functional ability at T2		−.38 (−.47; −.30)			−.64 (−.68; −.60)	
Functional ability at T3			−.57 (−.71; −.42)			−.80 (−.86; −.75)
Cognitive capacity at T1	−.04 (−.11; .04)			.11 (.08; .14)		
Cognitive capacity at T2		.03 (−.01; .08)			.16 (.14; .19)	
Cognitive capacity at T3			.10 (.02; .18)			.21 (.17; .24)
Neuropsychiatric symptoms at T1	−.04 (−.44; .37)			−.76 (−.91; −.60)		
Neuropsychiatric symptoms at T2		−.40 (−.63; −.17)			−.85 (−1.00; −.70)	
Neuropsychiatric symptoms at T3			−.86 (−1.28; −.43)			−1.12 (−1.32; −.92)
Relationship quality rated by people with dementia at T1	−.07 (−.33; .19)			.05 (−.05; .16)		
Relationship quality rated by people with dementia at T2		−.04 (−.17; .10)			.07 (−.02; .16)	
Relationship quality rated by people with dementia at T3			−.04 (−.31; .23)			.05 (−.11; .21)
Caregiver stress at T1	−.04 (−.16; .09)			−.29 (−.33; −.25)		
Caregiver stress at T2		−.13 (−.20; −.06)			−.30 (−.34; −.26)	
Caregiver stress at T3			−.24 (−.37; −.11)			−.36 (−.42; −.31)
Relationship quality rated by the caregiver at T1	.16 (−.06; .39)			.42 (.34; .50)		
Relationship quality rated by the caregiver at T2		.17 (.05; .29)			.54 (.47; .62)	
Relationship quality rated by the caregiver at T3			.12 (−.14; .37)			.62 (.51; .73)

Adjusted for age of the person with dementia, sex of the person with dementia, and diagnosis subtype.

## Discussion

This study explored for the first time whether and how involvement in everyday decision-making, as perceived by caregivers, changed in people with dementia over 2 years. The study also investigated how this change was associated with a range of personal characteristics of the person with dementia and caregiver, as well as cognitive capacity, functional ability, neuropsychiatric symptoms, relationship quality, and caregiver stress. Findings suggest that people with dementia were generally involved in everyday decision-making and this involvement only minimally decreased over time. According to their caregivers, those people with dementia who were female, single, with a university degree, and whose caregiver was younger were more involved in everyday decision-making compared to those without these characteristics. Those with frontotemporal dementia were less involved in everyday decision-making compared to those with Alzheimer’s disease. Better cognitive capacity, fewer functional difficulties, fewer neuropsychiatric symptoms, less caregiver stress, and better informant-rated relationship quality were consistently associated with higher involvement in everyday decision-making at baseline and follow-ups.

There was some variability in how involved people with dementia were in specific decisions. People with dementia were involved in infrequent but important decisions such as were to live. They were also very involved in certain daily and personal decisions such as where to go, when to get up, medical care, what to do in spare time, and expressing affections. Deciding where to live, when to go to bed and to get up have all been found to contribute to maintenance of routine, selfhood, and good quality of life in people with dementia (Bonds et al., 2021; Davis et al., 2017; Fetherstonhaugh et al., 2013; Menne et al., 2002, 2008, 2009; Menne & Whitlatch, 2007). However, people with dementia were less involved in decisions about other everyday personal activities such as visiting friends, where to go, and spending money. Although it may be reasonable for caregivers to take over decisions about how to spend money to prevent mismanagement of financial resources (Alzheimer Europe, 2020), people with dementia might be expected to maintain involvement in decisions regarding visiting friends and where to go. These activities may be fundamental for people with dementia to maintain friendships, interests, and hobbies, and general engagement with life.

The proportion of people with dementia involved in everyday decisions was lower for decisions about doing physical activity, having a pet, and engaging in religious and spiritual activities. Although being involved in these decisions may be very important for certain individuals and for their quality of life (Nuzum et al., 2020; Opdebeeck et al., 2021), other older people may lack interest in these areas, explaining why a smaller proportion of people with dementia was involved in these decisions.

People with dementia with a university degree were more involved in everyday decision-making compared to those with no educational qualifications. Greater education is related to better maintenance of cognitive abilities in older age and to slower cognitive deterioration among people with dementia (Mondini et al., 2016; Opdebeeck et al., 2016; Stern, 2009). Hence, it may be that people with a university education were more involved in decision-making activities due to better cognitive skills. Overall, our results suggest that strengthening education, for instance, through lifelong continuous education programs, may support better adjustment to the onset of dementia, including maintenance of involvement in decision-making.

In line with existing evidence, being female was associated with greater involvement in everyday decision-making (Menne & Whitlatch, 2007). The current sample included a majority of men with dementia who had a spousal caregiver. It may be that, as evidence suggests that men are generally less involved than women in everyday decisions regardless of having a diagnosis of dementia, their spouses or co-habiting partners think they know their preferences without having to directly ask about everyday decisions (Samsi & Manthorpe, 2013). In support of this reasoning, we found that involvement in decisions regarding what to wear, what food to buy, and what to eat at meals was markedly higher among women with dementia compared to men. People with dementia with older caregivers were also rated as having lower involvement in everyday decision-making, though this may be due to older caregivers primarily comprising spouses and spouses being more likely to take decisions for the person with dementia, whereas single people are more involved in everyday decision-making.

How involved people with dementia were in everyday decision-making slightly decreased over time; this result confirms longitudinal qualitative evidence showing that as dementia severity increases people with dementia become gradually less involved in everyday decision-making (Samsi & Manthorpe, 2013), though mean cognitive capacity scores showed only a gradual decline over the 2 years. More impaired cognition, increased functional difficulties, and more neuropsychiatric symptoms were related to having less involvement in everyday decision-making at all timepoints. It may be that caregivers gradually take more decisions on behalf of the person with dementia with the intent to decrease risk for the person with dementia (Alzheimer Europe, 2020; Stevenson et al., 2018). It may also be that caregivers simply do not know how to continue to involve people with dementia in everyday decision-making as dementia progresses. Wider availability of educational resources that inform caregivers about strategies to facilitate involvement in everyday decision-making, tailored to the stage of dementia, could prolong decision-making involvement in the person with dementia. Simplifying questions, using personalized visual aids or objects, and giving more time to formulate answers can all help to facilitate how involved the person with dementia is in everyday decision-making as dementia severity increases (Collins et al., 2022; Davis et al., 2017). Strategies designed to support functional ability in everyday activities in people with dementia, such as cognitive rehabilitation programs (Clare et al., 2019a, 2019b; Yates et al., 2019), may also maintain the involvement of people with dementia in everyday decision-making.

Higher levels of caregiver stress and lower relationship quality were also associated with less involvement in decision-making at all timepoints in the adjusted models. This links with previous evidence indicating that caregiver factors influence how caregivers appraise the abilities and functioning of the person with dementia (Martyr et al., 2019; Quinn et al., 2020). This suggests that making decisions on behalf of the person with dementia may increase caregiver stress, especially when caregivers are adult children or friends who may be less familiar with the daily preferences of the person with dementia compared to a spouse or co-habiting partner (Samsi & Manthorpe, 2013). Therefore, knowledge of the habits and preferences of the person with dementia appears essential to maintain involvement in those decisions that have always been important to the person. Post-diagnostic support may not only be important to equip caregivers with practical knowledge about how to continue involving the person with dementia in everyday decision-making despite dementia severity increasing, but may also provide psychological support and understanding of the emotional impact of taking some decisions on behalf of the person with dementia. However, there appears to be limited availability of adequate post-diagnostic support for people with dementia and their caregivers (van Horik et al., 2022). Finally, it may be that caregivers that are more stressed feel they have no time to involve the person with dementia in everyday decisions and that it is more practical to do tasks themselves.

### Strengths and Limitations

This study has some limitations that need to be acknowledged. The Decision-Making Involvement Scale was designed to be informant-rated only; therefore, there are no corresponding ratings made by the person with dementia, and information from the perspective of the person with dementia, or observational data concerning involvement in decision-making, was not collected. Findings may therefore be biased by level of caregiver stress, especially when the person with dementia is more cognitively impaired, has increased functional difficulties, and more neuropsychiatric symptoms (Kim et al., 2012; Martyr et al., 2019; Perren et al., 2006). It may be that those participants who dropped out over the 2 years of the study were in poorer health and less likely to be involved in everyday decision-making.

Two years was sufficient to detect a small change in everyday decision-making and cognitive and functional difficulties, but a longer follow-up could have elicited information concerning involvement in decisions around moving into care for more people with dementia as well as further understanding of decision-making as dementia severity increases. Nonetheless, this is one of the few studies exploring everyday decision-making in people with dementia over time and associations with a wide range of variables related to both the person with dementia and his/her caregiver.

## Conclusion

Overall, caregivers reported that people with dementia were somewhat involved in various aspects of everyday decision-making and this involvement declined only minimally over time. However, people with dementia with more functional and cognitive limitations, and/or whose caregivers are more stressed, may be at risk of being less involved in everyday decisions. Supporting people with dementia to remain involved in everyday decision-making as cognitive and functional difficulties increase may help maintain a good quality of life and sense of independence. Educational resources that provide caregivers with strategies that facilitate involvement of the person with dementia in everyday decision-making, as well as psychological support for caregivers, could help facilitate involvement of the person with dementia in everyday decision-making.

## Supplemental Material

Supplemental Material - Longitudinal Predictors of Informant-Rated Involvement of People with Dementia in Everyday Decision-Making: Findings from the IDEAL Program Click here for additional data file. Supplemental Material for Longitudinal Predictors of Informant-Rated Involvement of People with Dementia in Everyday Decision-Making: Findings from the IDEAL Program by Serena Sabatini, Anthony Martyr, Laura D. Gamble, Rachel Collins, Fiona E. Matthews, Robin G. Morris, Jennifer M. Rusted, Claire Pentecost, Catherine Quinn, and Linda Clare on behalf of the IDEAL study team in Journal of Applied Gerontology
