# Supplementary material for: Longitudinal Predictors of Informant-Rated Involvement of People with Dementia in Everyday Decision-Making: Findings from the IDEAL Program
Source: J Appl Gerontol. 2022 Oct 4;42(2):290–301. doi: 10.1177/07334648221128558 (PMC9841822; doi:10.1177/07334648221128558)
Supplement: Supplemental Material - Longitudinal Predictors of Informant-Rated Involvement of People with Dementia in Everyday Decision-Making: Findings from the IDEAL Program [file sj-pdf-1-jag-10.1177_07334648221128558.pdf]

Supplementary Table 1. Number and proportion of participants endorsing each response option for each item of the decision-making involvement scale

	Baseline	12-month follow-up	24-month follow-up
	n (%)		
How involved is your relative/friend in decisions about what to spend his/her money on?			
Not at all involved	98 (8.4)	120 (12.7)	115 (16.4)
A little involved	277 (23.8)	227 (24.1)	199 (28.4)
Fairly involved	313 (26.8)	226 (24.0)	159 (22.7)
Very involved	478 (41.0)	369 (39.2)	227 (32.4)
How involved is your relative/friend in decisions about visiting with friends?			
Not at all involved	107 (9.2)	115 (12.2)	130 (18.6)
A little involved	273 (23.5)	218 (23.1)	173 (24.8)
Fairly involved	374 (31.3)	287 (30.5)	190 (27.2)
Very involved	418 (36.0)	322 (34.2)	205 (29.4)
How involved is your relative/friend in decisions about what foods to buy?			
Not at all involved	164 (14.1)	189 (20.0)	190 (27.2)
A little involved	335 (28.8)	261 (27.7)	189 (27.1)
Fairly involved	300 (25.8)	244 (25.9)	159 (22.8)
Very involved	366 (31.4)	249 (26.4)	160 (22.9)
How involved is your relative/friend in decisions about when to go to bed?			
Not at all involved	42 (3.6)	40 (4.3)	46 (6.6)
A little involved	94 (8.1)	112 (12.0)	114 (16.3)
Fairly involved	265 (22.7)	210 (22.4)	164 (23.4)
Very involved	765 (65.6)	575 (61.4)	376 (53.7)
How involved is your relative/friend in decisions about when to get up?			
Not at all involved	51 (4.4)	58 (6.2)	60 (8.6)
A little involved	125 (10.7)	133 (14.2)	112 (16.1)
Fairly involved	261 (22.4)	219 (23.3)	183 (26.3)

Very involved	727 (62.5)	530 (56.4)	342 (49.1)
How involved is your relative/friend in decisions about what to do in his/her spare time?			
Not at all involved	54 (4.6)	68 (7.2)	74 (10.6)
A little involved	182 (15.6)	172 (18.3)	157 (22.4)
Fairly involved	349 (29.9)	305 (32.5)	207 (29.6)
Very involved	581 (49.8)	394 (42.0)	262 (37.4)
How involved is your relative/friend in decisions about being physically active?			
Not at all involved	114 (9.8)	114 (12.1)	116 (16.6)
A little involved	227 (19.5)	206 (21.9)	176 (24.2)
Fairly involved	327 (28.1)	286 (30.4)	186 (26.7)
Very involved	497 (42.7)	334 (35.5)	220 (31.5)
How involved is your relative/friend in decisions about participating in religious/spiritual activities?			
Not at all involved	525 (45.3)	447 (47.7)	327 (46.9)
A little involved	129 (11.1)	84 (9.0)	88 (12.6)
Fairly involved	133 (11.5)	100 (10.7)	76 (10.9)
Very involved	372 (32.1)	306 (32.7)	206 (29.6)
How involved is your relative/friend in decisions about expressing affection?			
Not at all involved	58 (5.0)	66 (7.0)	59 (8.5)
A little involved	219 (19.0)	204 (21.8)	177 (25.6)
Fairly involved	346 (30.1)	253 (27.0)	186 (26.9)
Very involved	528 (45.9)	414 (44.2)	270 (39.0)
How involved is your relative/friend in decisions about having a pet?			
Not at all involved	297 (25.8)	289 (31.1)	234 (33.6)
A little involved	67 (5.8)	67 (7.2)	52 (7.5)
Fairly involved	135 (11.7)	98 (10.6)	80 (11.5)
Very involved	651 (56.6)	474 (51.1)	331 (47.5)
How involved is your relative/friend in decisions about what to eat at meals?			
Not at all involved	120 (10.3)	122 (13.0)	109 (15.5)

A little involved	277 (23.8)	270 (28.7)	211 (39.1)
Fairly involved	362 (31.1)	277 (29.4)	199 (28.4)
Very involved	407 (34.9)	273 (29.0)	183 (26.1)
How involved is your relative/friend in decisions about choosing places to go?			
Not at all involved	98 (8.4)	110 (11.7)	103 (14.7)
A little involved	288 (24.8)	256 (11.7)	214 (30.5)
Fairly involved	406 (35.0)	306 (32.5)	195 (27.8)
Very involved	369 (31.8)	269 (28.6)	190 (27.1)
How involved is your relative/friend in decisions about what clothes to wear?			
Not at all involved	48 (4.1)	72 (7.6)	79 (11.3)
A little involved	181 (15.5)	188 (20.0)	165 (23.5)
Fairly involved	318 (15.5)	231 (24.5)	173 (24.6)
Very involved	619 (53.1)	451 (47.9)	285 (40.6)
How involved is your relative/friend in decisions about choosing where to live?			
Not at all involved	108 (9.3)	157 (16.9)	171 (24.7)
A little involved	98 (8.5)	77 (8.3)	64 (9.2)
Fairly involved	168 (14.5)	121 (13.0)	91 (13.1)
Very involved	783 (67.7)	576 (61.9)	367 (53.0)
How involved is your relative/friend in decisions about getting medical care?			
Not at all involved	68 (5.8)	121 (12.9)	124 (17.7)
A little involved	167 (14.3)	151 (16.1)	134 (19.1)
Fairly involved	338 (29.0)	265 (28.2)	174 (24.9)
Very involved	593 (50.9)	404 (42.9)	268 (38.3)

Supplementary Table 2. Number and proportion of men and women endorsing each response option for each item of the decision-making involvement scale

	Men	Women
	n (%)	
How involved is your relative/friend in decisions about what to spend his/her money on?		
Not at all involved	64 (9.3)	34 (7.1)
A little involved	185 (26.9)	92 (19.3)
Fairly involved	180 (26.2)	133 (27.8)
Very involved	259 (37.7)	219 (45.8)
How involved is your relative/friend in decisions about visiting with friends?		
Not at all involved	59 (8.6)	48 (10.1)
A little involved	193 (28.1)	80 (16.9)
Fairly involved	225 (32.7)	139 (29.3)
Very involved	211 (30.7)	207 (43.7)
How involved is your relative/friend in decisions about what foods to buy?		
Not at all involved	141 (20.5)	23 (4.8)
A little involved	227 (33.0)	108 (22.6)
Fairly involved	179 (26.1)	121 (25.3)
Very involved	140 (20.4)	226 (47.3)
How involved is your relative/friend in decisions about when to go to bed?		
Not at all involved	30 (4.4)	12 (2.5)
A little involved	70 (10.2)	24 (5.0)
Fairly involved	161 (23.4)	104 (21.8)
Very involved	427 (62.1)	338 (70.7)
How involved is your relative/friend in decisions about when to get up?		
Not at all involved	37 (5.4)	14 (2.9)
A little involved	86 (12.5)	39 (8.2)
Fairly involved	161 (23.4)	100 (21.0)

Very involved	404 (58.7)	67.9)
How involved is your relative/friend in decisions about what to do in his/her spare time?		
Not at all involved	39 (5.7)	15 (3.1)
A little involved	121 (17.6)	61 (12.8)
Fairly involved	213 (31.0)	136 (28.5)
Very involved	315 (45.8)	266 (55.7)
How involved is your relative/friend in decisions about being physically active?		
Not at all involved	73 (10.6)	41 (8.6)
A little involved	153 (22.3)	74 (15.5)
Fairly involved	186 (27.1)	141 (29.5)
Very involved	275 (40.0)	222 (46.4)
How involved is your relative/friend in decisions about participating in religious/spiritual activities?		
Not at all involved	340 (49.6)	185 (39.1)
A little involved	85 (12.4)	44 (9.3)
Fairly involved	69 (10.1)	64 (13.5)
Very involved	192 (28.0)	180 (38.1)
How involved is your relative/friend in decisions about expressing affection?		
Not at all involved	44 (7.5)	14 (3.0)
A little involved	143 (21.0)	76 (16.1)
Fairly involved	222 (32.7)	124 (26.3)
Very involved	271 (39.9)	257 (54.6)
How involved is your relative/friend in decisions about having a pet?		
Not at all involved	170 (25.0)	127 (27.0)
A little involved	45 (6.6)	22 (4.7)
Fairly involved	83 (12.2)	52 (11.1)
Very involved	382 (56.2)	269 (57.2)
How involved is your relative/friend in decisions about what to eat at meals?		
Not at all involved	101 (14.68)	19 (4.0)

A little involved	200 (29.1)	77 (16.1)
Fairly involved	217 (31.5)	145 (30.3)
Very involved	170 (24.7)	237 (49.6)
How involved is your relative/friend in decisions about choosing places to go?		
Not at all involved	71 (10.4)	27 (5.7)
A little involved	183 (26.7)	105 (22.1)
Fairly involved	247 (36.1)	159 (33.4)
Very involved	184 (26.9)	185 (38.9)
How involved is your relative/friend in decisions about what clothes to wear?		
Not at all involved	44 (6.4)	4 (.8)
A little involved	145 (21.1)	36 (7.5)
Fairly involved	227 (33.0)	91 (19.0)
Very involved	272 (39.5)	347 (72.6)
How involved is your relative/friend in decisions about choosing where to live?		
Not at all involved	75 (11.0)	33 (4.0)
A little involved	66 (9.7)	32 (6.8)
Fairly involved	99 (14.5)	69 (14.6)
Very involved	444 (64.9)	339 (71.7)
How involved is your relative/friend in decisions about getting medical care?		
Not at all involved	51 (7.4)	17 (3.6)
A little involved	116 (16.9)	51 (10.7)
Fairly involved	193 (28.1)	145 (30.3)
Very involved	328 (47.7)	265 (55.4)
